# The Effect of Component Defects on the Performance of Perovskite Devices and the Low-Cost Preparation of High-Purity PbI_2_

**DOI:** 10.3390/molecules29163810

**Published:** 2024-08-11

**Authors:** Boyu Dong, Yuhan Xie, Yongbing Lou

**Affiliations:** School of Chemistry and Chemical Engineering, Southeast University, Nanjing 211189, China; 220213205@seu.edu.cn (B.D.); 220213210@seu.edu.cn (Y.X.)

**Keywords:** perovskite solar cells, stoichiometry, low-cost fabrication, high-purity PbI_2_

## Abstract

The efficiency and reproducibility of perovskite solar cells (PSCs) are significantly influenced by the purity of lead iodide (PbI_2_) in the raw materials used. Pb(OH)I has been identified as the primary impurity generated from PbI_2_ in water-based synthesis. Consequently, a comprehensive investigation into the impact of Pb(OH)I impurities on film and device performance is essential. In this study, PbI_2_, with varying stoichiometries, was synthesized to examine the effects of different Pb(OH)I levels on perovskite device performance. The characterization results revealed that even trace amounts of Pb(OH)I impede the formation of precursor prenucleation clusters. These impurities also increase the energy barrier of the α-phase and facilitate the transition of the intermediate phase to the δ-phase. These effects result in poor perovskite film morphology and sub-optimal photovoltaic device performance. To address these issues, a cost-effective method for preparing high-stoichiometry PbI_2_ was developed. The formation of Pb(OH)I was effectively inhibited through several strategies: adjusting solution pH and temperature, modifying material addition order, simplifying the precipitation–recrystallization process, and introducing H_3_PO_2_ as an additive. These modifications enabled the one-step synthesis of high-purity PbI_2_. PSCs prepared using this newly synthesized high-stoichiometry PbI_2_ demonstrated photovoltaic performance comparable to those fabricated with commercial PbI_2_ (purity ≥ 99.999%). Our novel method offers a cost-effective alternative for synthesizing high-stoichiometry PbI_2_, thereby providing a viable option for the production of high-performance PSCs.

## 1. Introduction

Perovskite solar cells (PSCs) have emerged as a promising next-generation photovoltaic technology, characterized by low-cost production and remarkable power conversion efficiency (PCE). The rapid advancement in PCE, from 3.8% to 26.1%, underscores the significant potential of PSCs for large-scale industrial fabrication [1,2]. In terms of optoelectronic performance, PSCs have been demonstrated to be comparable to crystalline silicon cells [3]. These exceptional characteristics are attributed to the outstanding optoelectronic properties of perovskite materials, including an appropriate tunable bandgap [4], low exciton binding energy [5], excellent bipolar carrier transport [6], long carrier diffusion length [7], and high defect tolerance [8,9,10].

The development of efficient PSCs is contingent upon several factors, with the highly optimized morphology of perovskite thin films standing out as a critical element [11,12]. To enhance the quality and crystallinity of perovskite thin films, various methodologies have been employed by researchers. These approaches aim to prepare perovskite films with uniform morphology, high crystallinity, and pinhole-free surfaces. Strategies include the introduction of diverse additives [13,14,15,16,17], the refinement of deposition methods [18,19,20], the implementation of anti-solvent engineering [21], and the optimization of solvent engineering techniques [22,23,24]. Additionally, the stoichiometry of precursor components has been recognized as a crucial factor influencing efficiency [25]. Minor alterations in precursor solution stoichiometry have been observed to significantly impact the quality of perovskite layers, consequently affecting the PCE of PSCs. Researchers have focused on these fractional deviations, revealing their substantial influence on photovoltaic performance [7,26,27,28].

Lead iodide (PbI_2_), as a precursor raw material, is critically important for the photovoltaic performance of PSCs, yet this aspect is rarely discussed. The efficiency and reproducibility of PSCs are heavily dependent on the purity of raw materials, necessitating an in-depth investigation into the impact of impurities on thin-film and device performance. Numerous research institutions have discovered that commercially available PbI_2_, even with purities of ≥99.999%, contains insoluble impurities that significantly degrade the optoelectronic performance of PSCs [29]. Ross et al. [30] provided a comprehensive list of known impurities in perovskite reagents and noted that aqueous re-crystallization could effectively reduce impurity levels, although this process might introduce new impurities. The labeled purity of PbI_2_ often fails to accurately represent its actual stoichiometry due to the inclusion of Pb-based by-products, which are typically outlined in product specifications but not considered in the labeled purity. This discrepancy indirectly leads to a poor repeatability of perovskite photovoltaic performance across different laboratories. The primary impurity in hydrothermal synthesis or aqueous re-crystallization has frequently been demonstrated to be lead-hydroxyl-iodate (Pb(OH)I) [29,31,32,33]. Senevirathna et al. [34] identified Pb(OH)I as the main component responsible for insoluble impurities and reduced photovoltaic performance in PSCs. Despite the apparent clarity of dimethylformamide (DMF) solutions containing high-purity PbI_2_, trace amounts of Pb(OH)I may still be present. Surprisingly, research on the effects of trace Pb(OH)I on PSC performance has been largely overlooked.

Traditional PbI_2_ synthesis methods primarily include water-based synthesis and high-temperature diffusion synthesis [35]. The water-based synthesis method introduces unnecessary impurities, such as Pb(I_1−x_O_x_)_2_ and Pb(I_1−y_(OH)_y_)_2_ [29], while high-temperature synthesis can provide high-quality raw materials, but at a significantly higher cost [36]. Consequently, the water-based synthesis method offers more advantages during the commercialization of PbI_2_ due to its cost-efficiency. As previously mentioned, the main impurity in water-based synthesis is Pb(OH)I, emphasizing the need to investigate the impact of trace amounts of Pb(OH)I on perovskite device performance. In the large-scale industrialization of PSCs, low-cost and high-purity raw materials are of utmost importance. The high-purity PbI_2_ available on the market is expensive, limiting its use to research purposes and severely restricting commercial production. Researchers commonly assume the inherent procedural step of the “direct precipitation of PbI_2_ crude products from lead-containing and iodine-containing solutions” when using aqueous synthesis methods to seek pathways for synthesizing high-purity PbI_2_. The primary focus has been on purifying PbI_2_ crude products to obtain high-purity PbI_2_, which significantly increases manufacturing costs. However, the feasibility of synthesizing high-purity PbI_2_ directly from reactants in a one-step process has been largely overlooked. Therefore, the development of a low-cost, one-step synthetic pathway for high-purity PbI_2_ is imperative.

In this paper, the impact of trace Pb(OH)I on the photovoltaic performance of PSCs was investigated by synthesizing PbIx with varying stoichiometric ratios under different reaction conditions. The results demonstrate that Pb(OH)I plays a crucial role in the formation of intermediate phases within precursor solutions. This participation increases the energy barrier for phase transformation and promotes δ-phase generation, resulting in a substantial number of defects in the thin film. Consequently, non-radiative recombination occurs, leading to energy loss. Upon elucidating the influence of Pb(OH)I, the primary impurity in this setting, on device performance and its formation mechanism during aqueous synthesis, a novel method for synthesizing high-purity PbI_2_ was developed. This method effectively inhibits Pb(OH)I formation through several strategic modifications: altering the solution environment, adjusting the order of reactant addition, streamlining the precipitation and re-crystallization purification processes, and incorporating H_3_PO_2_ additives. These modifications synergistically suppress the formation of Pb(OH)I. Notably, devices prepared using PbI_1.995_ exhibited a performance comparable to those utilizing high-purity PbI_2_ (≥99.999% sourced from Polymer). Our present work not only elucidates the impact of trace Pb(OH)I on PSC performance, but also presents a practical, cost-effective raw material solution for the large-scale commercialization of perovskite photovoltaics.

## 2. Results and Discussion

The pH value of the solution significantly influences the generation of Pb(OH)I. Relevant research has indicated that in weakly acidic environments with pH values below 6, PbI_2_ can be obtained without forming the Pb(OH)I phase [37]. However, our findings reveal that PbI_2_ synthesized in solutions with pH values lower than 5 still contained insoluble Pb(OH)I impurities when dissolved in DMF solution, as illustrated in Figure S1a. DMF solutions of PbI_2_ appeared clear in various batches, as shown in Figure S1b. Nevertheless, the Pb and I proportions varied between batches when determining the stoichiometric ratio, implying the difficulty in directly observing micro-impurities in PbI_2_ prepared this way. These observations suggest that Pb(OH)I cannot be completely eliminated solely by controlling the solution’s pH value during the water-based synthesis process. Furthermore, the minute quantities of Pb(OH)I present in PbI2 prepared through this method are challenging to detect intuitively and are often overlooked in PSC performance studies. This oversight underscores the need for more effective detection methods and a deeper understanding of the role of trace impurities in PSC performance.

**Figure 1:**
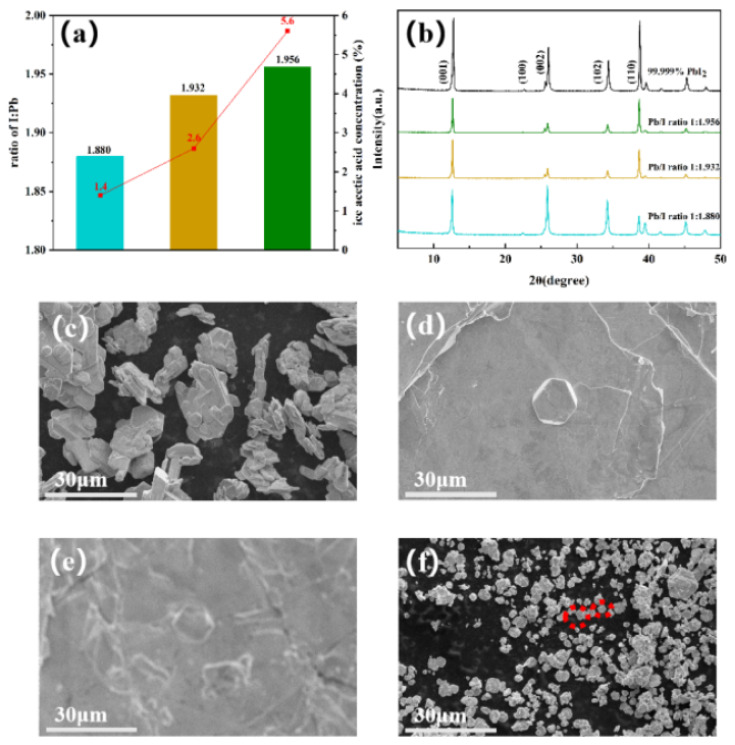
(**a**) The stoichiometry of different PbI_2_ samples; (**b**) XRD patterns of commercial PbI_2_ and different stoichiometric PbI_2_; SEM images (**c**–**f**) of commercial PbI_2_ and different stoichiometric PbI_2_; (**c**) commercial PbI_2_; (**d**) PbI_1.956_; (**e**) PbI_1.932_; (**f**) PbI_1.880_. The red circle indicates the needle-shaped crystal structure Pb(OH)I.

Previous reports have confirmed that a higher pH value of the solution results in the formation of Pb(OH)I. Based on this, we synthesized PbI_2_ with varying amounts of Pb(OH)I by adjusting the solution’s pH value. High-purity PbI_2_ (purity of ≥99.999% purchased from Polymer) was taken as a control group. Since the determination of commercial PbI_2_ purity does not consider lead-based impurities, this study utilized the I/Pb molar ratio to indicate PbI_2_ purity. Aqueous re-crystallization effectively removes OAc and metal impurities; however, it remains challenging to remove residual Pb(OH)I. The theoretical stoichiometry of pure PbI_2_ should exhibit a Pb/I ratio of 1:2. Determining the stoichiometry of PbI_2_ essentially serves as an indirect measurement of Pb(OH)I content. Multiple methods have been employed by researchers to conduct stoichiometric measurements of PbI_2_ [7,29,30], such as XPS, RBS, and ICP-OES. XPS, a surface characterization technique, can be adopted to obtain the atomic stoichiometric ratio of a sample’s surface. However, X-rays can cause the decomposition of PbI_2_, resulting in the strengthening of the Pb signal peak [38]. RBS introduces errors at a rate of 4% due to uncertainties in ion-beam stopping power. While ICP-OES effectively quantifies multiple elements, it is unsuitable for hydroxide testing and has a 5% error rate [30]. These instruments exhibit excessively large systematic errors for detecting minute amounts of Pb(OH)I in PbI_2_. To determine PbI_2_ stoichiometry, a complexometric titration scheme was designed to measure Pb(OH)I impurity content (Figure S2). The titration error can be controlled within ±0.1%. The stoichiometric ratios of different PbI_2_ samples are presented in Figure 1a. Additionally, the Pb content in PbI_2_ samples with different stoichiometric ratios was determined by ICP-OES, as shown in Table S1. The mass fraction of Pb in PbI_2_ decreases from 47.228% to 46.748%, indicating an increase in the anion proportion. As the Pb(OH)I impurity content decreases and the PbI_2_ content increases, a higher I/Pb stoichiometric ratio is observed. This suggests that the change in Pb content in the samples corresponds with the measured PbI_2_ stoichiometric ratio, indirectly reflecting the accuracy of the titration scheme.

The crystal structure and phase composition of PbI_2_ samples with varying stoichiometries were investigated using X-ray diffraction (XRD) patterns (Figure 1b). Both the water-synthesized PbI_2_ and commercial PbI_2_ present similar patterns, namely hexagonal PbI_2_ with a P-3m1 space group. A progressive decrease in PbI_2_ stoichiometry is observed to correlate with a reduction in peak intensities for the (001) and (110) crystal planes. This reduction indicates a decrease in grain size and crystallinity. Notably, the lattice orientation of the PbI_1.880_ crystals differs significantly from the other PbI_2_ crystals with higher I/Pb ratios. The impact of Pb(OH)I content on PbI_2_ morphology was subsequently examined. Commercial PbI_2_ presents continuous hexagonal structures with smooth powder particles of varying size (Figure 1c). Smaller hexagonal structures are observed to stack upon larger layered structures, with average sizes exceeding 10 μm and well-defined outlines. The morphology of PbI_1.956_ shows alterations, including reduced crystal size and irregular hexagonal forms (Figure 1d). As shown in Figure 1e, the crystal structure of PbI_1.932_ deforms, and the layered structures become fragmented with apparent destruction. The hexagonal dimension of the film is approximately 10 μm, suggesting a reduction in size, with a blurred outline and rough surface. Moreover, the PbI_1.880_ crystal particles are fragmented with an average size of approximately 2 to 3 μm (Figure 1f). As the stoichiometric ratio decreases, we note that the hexagonal crystal structure of PbI_2_ becomes increasingly irregular, with gradual particle size reduction and blurred contours. The hydrothermal synthesis of PbI_1.880_ results in deformed structures and needle-shaped crystals corresponding to the morphology of Pb(OH)I (Figure S3). Despite the presence of pronounced needle-shaped Pb(OH)I impurities in the scanning electron microscopy (SEM) image of PbI_1.880_, no additional impurity peaks are detected in its XRD pattern. This observation suggests a low impurity content of Pb(OH)I, with weak scattering intensity preventing signal detection. We infer that Pb(OH)I may influence the surface energy of PbI_2_ crystals, leading to the observed evolution of particle crystal morphology in the samples. These findings underscore the critical importance of inhibiting Pb(OH)I formation and maintaining the stoichiometric balance between I^−^ and Pb^2+^ ions to preserve the stability of crystal structures.

**Figure 2:**
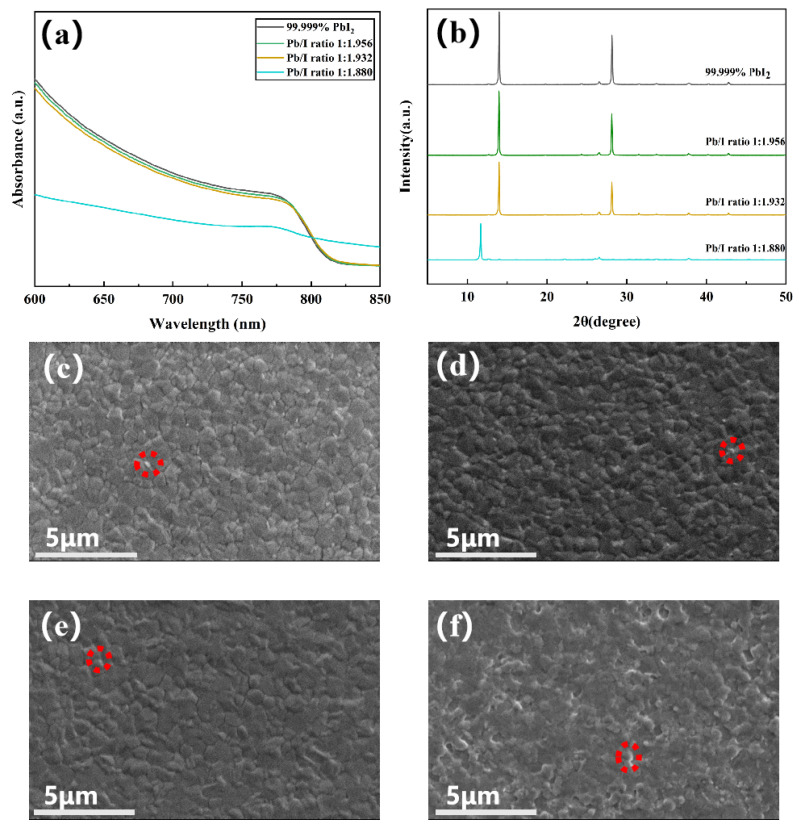
(**a**) XRD patterns of perovskite thin films prepared from commercial PbI_2_ and different stoichiometric PbI_2_; (**b**) ultraviolet absorption spectra of perovskite thin films prepared from commercial PbI_2_ and different stoichiometric PbI_2_; SEM images (**c**–**f**) of perovskite thin films prepared from commercial PbI_2_ and different stoichiometric PbI_2_; (**c**) commercial PbI_2_; (**d**) PbI_1.956_; (**e**) PbI_1.932_; (**f**) PbI_1.880_. Unreacted PbI_2_ marked in red circle.

The effect of PbI_2_ stoichiometry on the resultant perovskite film was then investigated using Cs_0.05_FA_0.95_PbI_3_ as a model system. We found that, during the annealing process, PbI_1.880_ rapidly turned into a non-optically active yellow phase, proving that the intermediate phases of the perovskite films prepared from PbI_1.880_ were extremely unstable (Figure S4a,b). UV-vis spectra of the perovskite films are shown in Figure 2a. A correlation is observed between decreasing PbI_2_ stoichiometry and a reduction in absorption peak intensity. The presence of trace impurities results in minimal differences in absorbance. However, the ultraviolet absorption peak of the perovskite film prepared from PbI_1.880_ is significantly suppressed, corresponding to its transformation into the yellow phase. Perovskite films synthesized from PbI_2_ precursors with different stoichiometries were characterized by XRD, as shown in Figure 2b. An increase in PbI_2_ stoichiometry is found to correlate with an intensification of signal strength, indicating enhanced film quality and crystallinity. The diffraction peak at 2θ = 14° corresponds to the characteristic (110) crystal plane of Cs_0.05_FA_0.95_PbI_3_. The characteristic peaks corresponding to the unreacted PbI_2_ are the scattered peaks around 2θ = 12.6°, marked with an asterisk, revealing that the precursors were not entirely transformed into the perovskite films (Figure S5). A magnified XRD pattern of the film prepared from PbI_1.880_ reveals diffraction peaks corresponding to the non-optically active δ-phase, PbI_2_ impurity, and the characteristic (110) crystal plane, from left to right. This observation implies a predominant transformation of perovskites from the α-phase to the δ-phase. Furthermore, a reduction in stoichiometry is associated with decreased crystallinity and quality, as well as a transition to the δ-phase. These findings suggest that the presence of Pb(OH)I inhibits the reaction of PbI_2_, promoting the film’s transition to the δ-phase. The absence of significant peak shifts in the perovskite films indicates that Pb(OH)I does not incorporate into the perovskite lattice but rather exerts its influence on the film surface and grain boundaries.

SEM imaging revealed a deterioration in perovskite film quality with decreasing PbI_2_ stoichiometry, manifesting in the form of rougher surfaces, lower degrees of crystallinity, and more heterogeneous morphologies (Figure 2c–f). The white particles observed on the surfaces of the perovskite films are identified as unreacted PbI_2_. The perovskite film prepared from PbI_1.880_ contains a substantial amount of residual PbI_2_, demonstrating that Pb(OH)I negatively affects the stability of the intermediate phase of the perovskite film, thereby compromising its overall quality.

**Figure 3:**
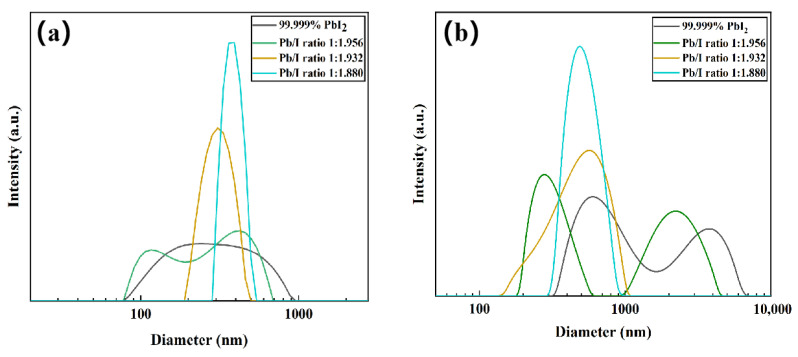
(**a**) DLS spectra of commercial PbI_2_ and different stoichiometric PbI_2_ in DMF solution; (**b**) DLS spectra of perovskite precursor solution prepared from commercial PbI_2_ and different stoichiometric PbI_2_.

The formation of a uniform perovskite film with high crystallinity is intricately linked to the composition and properties of the precursor in the perovskite precursor solution [39]. The size and distribution of the crystal nuclei in the precursor solution significantly influence the film’s grain growth mechanism. Evenly distributed crystal nuclei can result in uniform film grain size. Separate solutions of PbI_2_ in DMF and perovskite precursor in DMF/DMSO (9:1 volume ratio) were prepared. Dynamic light scattering (DLS) was employed to investigate the relationship between PbI_2_ stoichiometry and the size distribution of PbI_2_ colloids or precursor colloids, as well as the composition and properties of the perovskite precursor solution. As the stoichiometry decreases, the average size of PbI_2_ increases, as shown in Figure 3a. This phenomenon is primarily attributed to the coordination between N atoms in the DMF solvent and Pb^2+^ ions in the material. DMF molecules exfoliate PbI_2_ through defect points on the crystal faces of (001) and (101) [40]. However, Pb(OH)I hinders this coordination reaction. In contrast, PbI_1.880_ crystals exhibit a narrower colloidal size distribution with a smaller range of sizes. The precursor distribution is generally uneven (Figure 3b), consistent with the findings of Chao et al. [41]. Furthermore, as the I/Pb ratio decreases, the DLS peak center shifts leftward, indicating a reduction in the average crystal nucleus size and a wider size distribution. This decrease in average size suggests that Pb(OH)I impedes the formation of pre-nucleation clusters [42]. Even a minute amount of Pb(OH)I significantly interferes with crystallization, increasing the energy barrier for nucleation during crystallization. This inhibits rapid nucleation, resulting in the generation of perovskite films with uneven degrees of crystallinity. This trend is also observed in the perovskite precursor solution prepared from PbI_1.880_. Although the precursor solution contains crystal nuclei of uniform sizes, the distribution of large particle diameters is not reflected. This observation indicates that impurities inhibit the formation of pre-nucleation clusters. It is hypothesized that smaller PbI_2_ colloids are more conducive to participating in the precursor reaction, thus promoting the formation of α-phase intermediates and prenucleation clusters. Conversely, larger PbI_2_ colloids, affected by Pb(OH)I, and with a relatively smaller contact area, are less favorable for prenucleation cluster formation. In conclusion, the characteristics of PbI_2_ crystals influence the size distribution of PbI_2_ colloids, which subsequently affects the size distribution of PbI_2_ precursor colloids and the dimensions of prenucleation clusters. This intricate relationship plays a crucial role in determining the final properties of the perovskite film.

**Figure 4:**
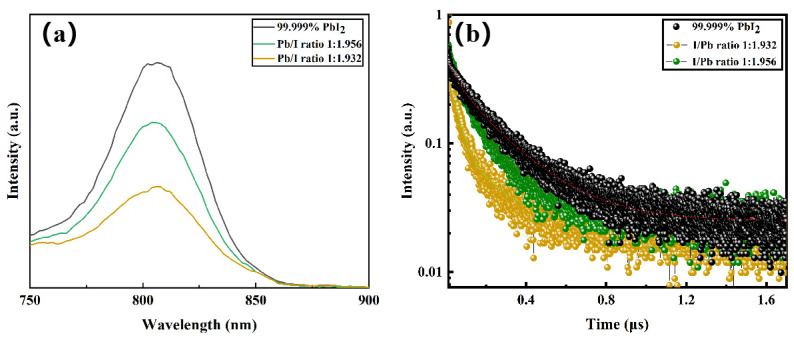
(**a**) PL spectra of perovskite thin films prepared from commercial PbI_2_ and different stoichiometric PbI_2_; (**b**) TRPL spectra of perovskite thin films prepared from commercial PbI_2_ and different stoichiometric PbI_2_.

To investigate the impact of minute amounts of Pb(OH)I on perovskite films’ defect properties, photo-luminescence (PL) and time-resolved photo-luminescence (TRPL) spectroscopy tests were conducted on films prepared from stoichiometric PbI_2_ with x ≥1.932. Films prepared from PbI_1.880_ exhibited apparent yellow phases and were excluded from the analysis. The perovskite films exhibited a strong fluorescence peak at 808 nm, corresponding to the position of the photoluminescence peak observed in previous UV-vis spectra (Figure 4a). A significant decrease in PL intensity is observed with decreasing stoichiometric ratio, indicating that trace Pb(OH)I amounts intensify non-radiative recombination defects, leading to reduced film PL intensity. The TRPL spectra revealed that improved PbI_2_ sample stoichiometry resulted in slower photo-luminescence attenuation speed (Figure 4b). The lifetime fitting analysis yielded carrier lifetimes of 224 ns, 148 ns, and 127 ns for perovskite films prepared from commercial PbI_2_, PbI_1.956_, and PbI_1.932_, respectively.

**Figure 5:**
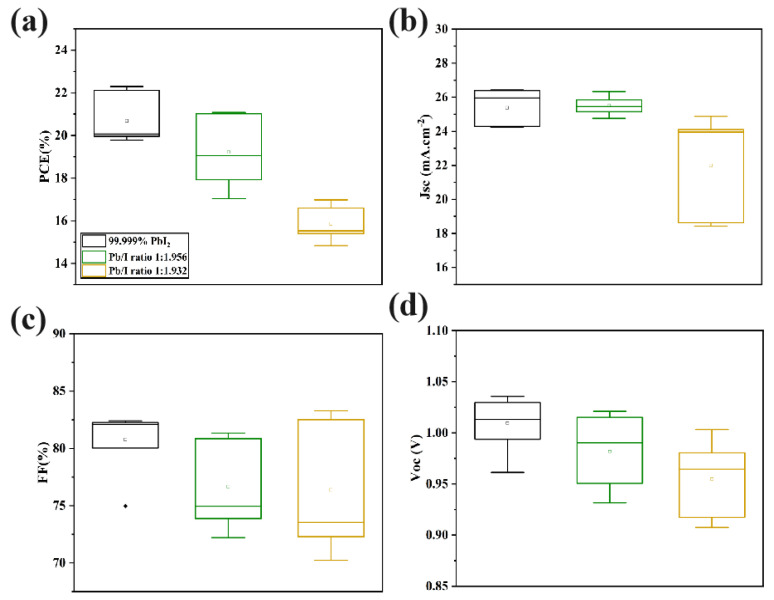
Box plots of perovskite devices: (**a**) PCE; (**b**) *Jsc*; (**c**) FF; (**d**) *Voc* prepared from commercial PbI_2_ and different stoichiometric PbI_2_. The structure was FTO/C−TiO_2_−SnO_2_/FA_0.95_Cs_0.05_PbI_3_/Spiro−MeOTAD/Ag.

PSCs with a structure of glass/FTO/C−TiO_2_−SnO_2_/FA_0.95_Cs_0.05_PbI_3_/Spiro−MeOTAD/Ag were fabricated to research the impact of trace amounts of Pb(OH)I on photovoltaic performance (Figure 5a–d). With reductions in stoichiometry, the PCE of the devices apparently declines, along with decreases in short-circuit current density (*Jsc*), fill factor (FF), and open-circuit voltage (*Voc*). The photovoltaic parameters of the champion PSC devices prepared from commercial PbI_2_ and PbI_2_ with varying stoichiometries under standard sunlight conditions are presented in Table 1. It can be observed that the accumulation of trace Pb(OH)I content correlates with deteriorating device performance.

To inhibit trace Pb(OH)I formation and synthesize low-cost and high-purity PbI_2_, understanding the production mechanism of Pb(OH)I impurity in water-based synthesis is crucial. The molar ratio of reactants significantly influences Pb(OH)I impurity content. Typically, KI and Pb(OAc)_2_ or Pb(NO_3_)_2_ are employed in a 2:1 mole ratio for water-based PbI_2_ synthesis [43]. During the synthesis and purification process, due to the insolubility of PbI_2_ and the particle agglomeration of the crude product, PbI_2_ needs to be dissolved in a high-temperature and acidic environment for a long time. In the hydrothermal solution, I^−^ is susceptible to oxidation by oxygen, forming free iodine that combines with I^−^ to generate I_3_^−^, which further reduces the stoichiometric ratio of I^−^ and Pb^2+^. Incompatible I^−^ and Pb^2+^ dosages, coupled with strong interactions between ions and water molecules, cause water self-ionization [44], resulting in a mixture containing PbI_2_ and trace amounts of Pb(OH)I. The re-crystallization process of PbI_2_ is identified as the primary factor contributing to the failure of the acidic environment to inhibit Pb(OH)I impurity formation. An innovative water-based synthesis method is employed to synthesize the high-stoichiometric-ratio PbI_2_ by inhibiting Pb(OH)I formation. Commercial PbI_2_ is retained as a control sample. The stoichiometry of the low-cost PbI_2_ and corresponding Pb amounts are presented in Table 2.

**Figure 6:**
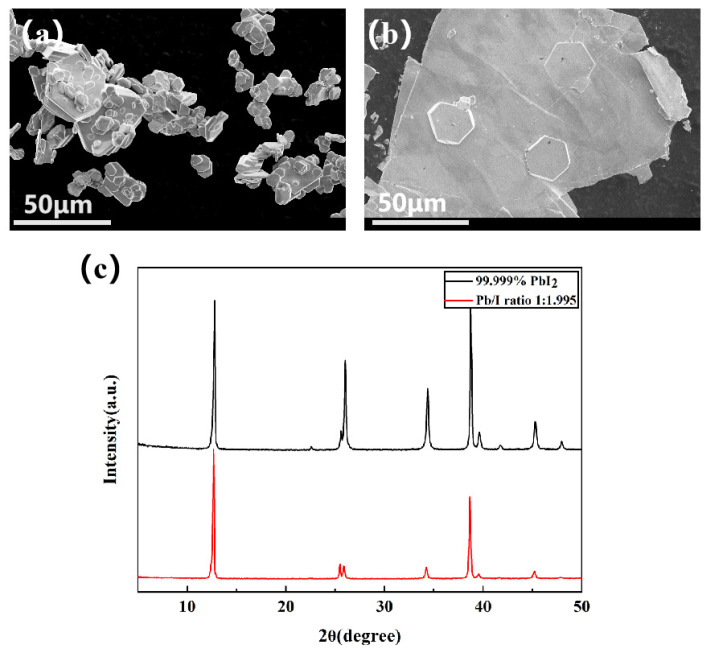
SEM images (**a**,**b**) of PbI_2_: (**a**) commercial PbI_2_; (**b**) PbI_1.995_. (**c**) XRD patterns of commercial PbI_2_ and PbI_1.995_.

SEM characterization was conducted on commercial PbI_2_ and PbI_1.995_. Both samples exhibited continuous layered structures and flat hexagonal crystal formations with larger crystal dimensions (Figure 6a,b). The XRD patterns of the commercial PbI_2_ and PbI_1.995_ samples synthesized via water-based methods presented similar modes (specifically, PbI_2_ with a P-3m1 space group) (Figure 6c). The results show that the structure and crystallinity of synthesized PbI_1.995_ are comparable to those of commercial PbI_2_, which suggests its potential as an alternative to high-purity, high-cost commercial PbI_2_.

**Figure 7:**
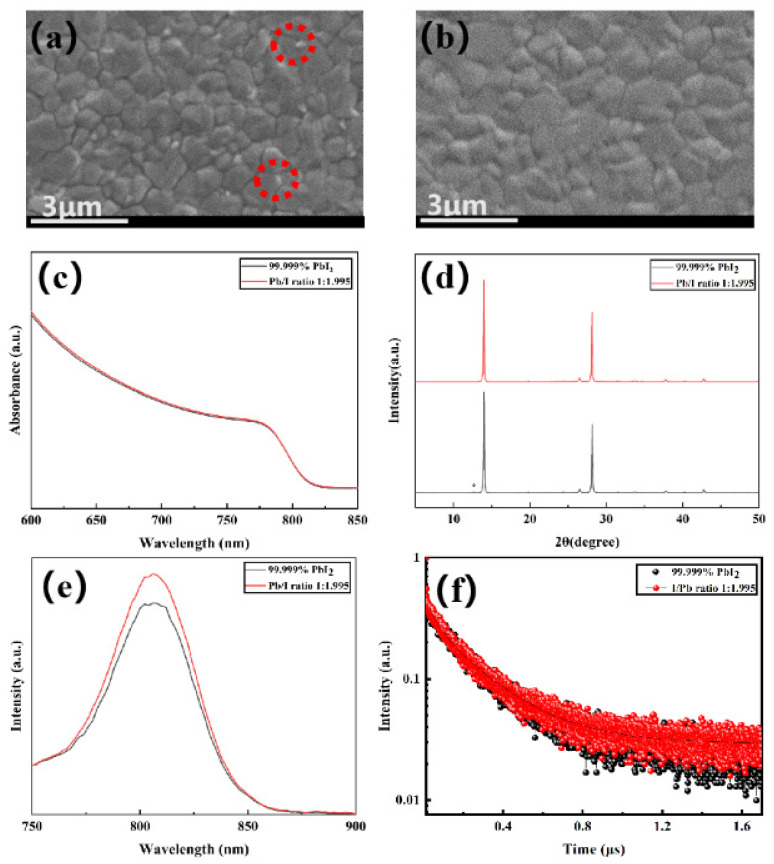
SEM images of perovskite films prepared from (**a**) commercial PbI_2_ and (**b**) PbI_1.995_; (**c**) ultraviolet absorption spectra of perovskite thin films; (**d**) XRD patterns of perovskite thin films; (**e**) PL spectra of perovskite thin films; (**f**) TRPL spectra of perovskite thin films. Unreacted PbI_2_ is marked in red circles and the peak indicated by the asterisk is the characteristic peak of PbI_2_ impurity.

To verify the enhancement in film quality using this strategy, perovskite films corresponding to commercial PbI_2_ and PbI_1.995_ were synthesized. SEM images revealed that the grain size of the perovskite film prepared from PbI_1.995_ was comparable to that of commercial PbI_2_, with no detectable residual PbI_2_ detected at the grain boundaries (Figure 7a,b). Residual PbI_2_ in perovskite films decomposes under prolonged photo-thermal conditions, producing Pb that acts as a center for non-radiative recombination. This process also induces additional related deep-level defects, negatively impacting the performance and stability of devices. Therefore, we believe that Pb_1.995_ has the potential to synthesize high-performance devices. The UV-vis spectra showed that the absorbance of the perovskite film prepared with PbI_1.995_ is slightly higher than that of the film prepared from commercial PbI_2_, exhibiting that it possesses superior light absorption capability (Figure 7c). The patterns of XRD revealed that while the perovskite film prepared from commercial PbI_2_ showed an impurity peak of PbI_2_, the film prepared from PbI_1.995_ did not display miscellaneous peaks of PbI_2_, suggesting the complete conversion of the PbI_1.995_ precursor into the perovskite film (Figure 7d and Figure S6). In addition, the PL intensity of the perovskite film prepared from PbI_1.995_ increased significantly (Figure 7e). The TRPL spectra demonstrated a slower photo-luminescence attenuation for the PbI_1.995_-derived perovskite film. Through lifetime fitting, we obtained that the carrier lifetime of perovskite film prepared from PbI_1.995_ is 229 ns. These results indicate that with the increase in the carrier lifetime of perovskite films, there is a reduction in trap density or a decrease in surface recombination. Consequently, carrier mobility is enhanced and the non-radiative recombination rate is reduced.

**Figure 8:**
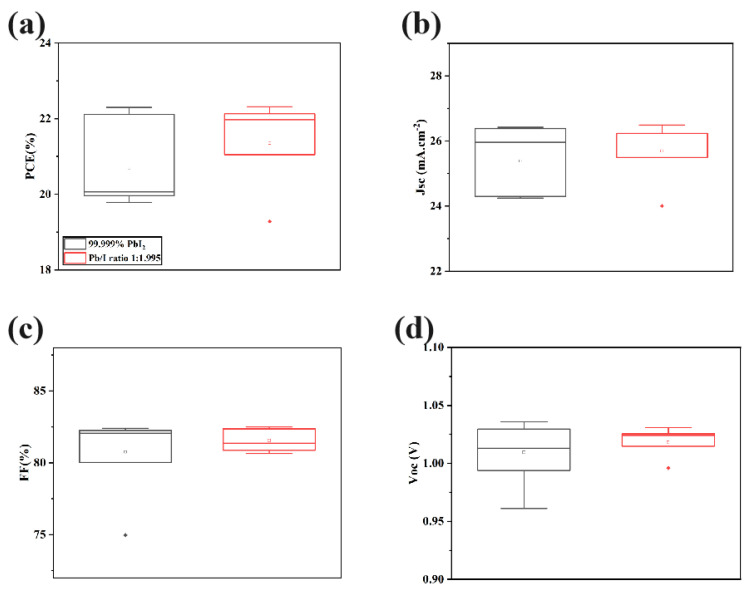
Box plots of perovskite devices: (**a**) PCE; (**b**) *Jsc*; (**c**) FF; (**d**) *Voc* prepared from commercial PbI_2_ and PbI_1.995_. The structure was FTO/C−TiO_2_−SnO_2_/FA_0.95_Cs_0.05_PbI_3_/Spiro−MeOTAD/Ag.

The fabrication of PSC devices with a structure of glass/FTO/C−TiO_2_−SnO_2_/FA_0.95_Cs_0.05_PbI_3_/Spiro−MeOTAD/Ag was performed to demonstrate how the low-cost and high-stoichiometry PbI_2_ affects device performance using this strategy (Figure 8a–d). PSCs prepared from commercial PbI_2_ achieved a champion PCE of 22.295%, while those prepared from PbI_1.995_ reached a champion PCE of 22.315%. The optimal device efficiency of PbI_1.995_ is comparable to that of devices prepared from commercial PbI_2_, demonstrating the viability of this cost-effective alternative.

## 3. Materials and Methods

### 3.1. Materials

Ethanol (99.7%), deionized water, ethylenediamine tetraacetic acid disodium salt dihydrate (EDTA, 99.0%), isopropanol (99.7%), and lead acetate trihydrate (Pb(CH_3_COO)_2_·3H_2_O, 99.5%) were purchased from Sinopharm Chemical Reagent Co., Ltd. Lead iodide (99.999%), methylamine chloride (MACl, 99.5%), cesium iodide (CsI, 99.999%), formimidamide hydroiodide (FAI, 99.5%), 2,2′,7,7′-Tetrakis[*N*,*N*-di(4-methoxyphenyl)-amino]-9,9′-spirobi -fluorene (Spiro-OMeTAD, 99%), bis (trifluoromethane) sulfonimide lithium salt (Li-TFSI, 99.5%), and tetraoctylammonium bromide (98%) were purchased from Xi’an Polymer Light Technology Corp., Ltd. Acetic acid (99.5%), hypophoaphoeous acid (H_3_PO_2_,50 wt. % in H_2_O), potassium iodide (KI, 99%), tin chloride dihydrate (SnCl_2_·2H_2_O, 98%), methanol (99.5%), and 4-tert- butylpyridine (4-tBP, 98%) were purchased from Aladdin. Acetonitrile (99.8%), *N*,*N*-Dimethylformamide (DMF, 99.8%), dimethyl sulfoxide (DMSO, 99.7%), and chlorobenzene (99%) were purchased from J&K Scientific Ltd.

### 3.2. Experimental Methods

Water-based synthesis of PbI_2_ powder: Weigh 1.64 g of Pb(CH_3_COO)_2_·3H_2_O and 1.48 g of KI. Dissolve them separately in 5 mL deionized water, and filter solutions to remove insoluble impurities. Then, take 400 mL deionized water in a round-bottom flask and heat it to 90 °C. Add the aforementioned Pb(CH_3_COO)_2_ solution into the deionized water, and then add 40 mL of acetic acid solutions of different concentrations (glacial acetic acid/water volume ratios: 31/9, 5/3, 3/7, 3/17, respectively). Add the prepared KI solution and stir for about 10 s to form a solution system with different glacial acetic acid concentrations (1.4~6.9%). Pour the solution into a beaker to cool and crystallize. The solution will become clear and transparent. Add 5 mL of H_3_PO_2_ to one of the sample solutions containing 6.9% acetic acid concentration (glacial acetic acid/water volume ratio: 31/9) to synthesize the highest stoichiometric ratio of the test sample during the crystallization process. Centrifuge and filter the cooled solution; wash it four times with anhydrous ethanol to remove residual impurities such as H_3_PO_2_. Dry the cleaned PbI_2_ in a vacuum-drying oven at 70 °C for 12 h to obtain PbI_2_ powder with different stoichiometric ratios.

Determination of PbI_2_ stoichiometry: Weigh 0.2 g of PbI_2_ and dissolve it in 20 mL of deionized water. Add 5 mL of a pH ≈ 5.5 sodium acetate–acetic acid (NaAc-HAc) buffer solution and heat it in a water bath until the sample dissolves. Add 3 drops of 0.2% xylenol orange indicator. Titrate with a prepared 0.02 M disodium EDTA (ethylenediaminetetraacetic acid) solution until the solution changes from purple-red to bright yellow. Perform parallel measurements three times to determine the concentration of Pb^2+^. Weigh 0.2 g of PbI_2_ and dissolve it in 7 mL of 0.1 M EDTA. Add 10 mL of 12 M HCl and heat the solution to boiling. Add 2 mL of CHCl_3_ as an indicator, then titrate with 0.055 M KIO_3_ solution until the solution changes from orange-red to colorless. Perform parallel measurements three times to determine the concentration of I^−^.

Perovskite precursor solution preparation: In a glovebox, weigh 0.2005 g of PbI_2_, 0.0711 g of FAI, 0.0056 g of CsI, and 0.0088 g of MACl. Measure 2.7 mL of DMF and 0.3 mL of DMSO, and mix them to form a 9:1 volume ratio DMF/DMSO solution. Add all the weighed reactants to the DMF/DMSO solution and sonicate until fully dissolved to form the FA_0.95_Cs_0.05_PbI_3_ perovskite precursor solution.

### 3.3. Device Fabrication

The FTO glass is soaked in a 2% sodium hydroxide ethanol solution overnight and rinsed with deionized water and ethanol, and the remaining liquid is blown off with nitrogen gas. A 20 nm thick compact TiO_2_ layer is deposited on the FTO glass using the spray pyrolysis method. A 0.75% SnO_2_ solution is spin-coated onto the TiO_2_ layer at 4000 rpm for 30 s, annealed at 150 °C, and then treated with UV/ozone for 20 min. Then, 60 μL of the prepared precursor solution is dropped onto the surface and spin-coated at 5000 rpm with an acceleration of 1000 rpm/s² for 15 s. Approximately 3 s before the end of spin-coating, 250 μL of chlorobenzene is added as an anti-solvent to form the perovskite layer, which is then annealed at 150 °C for 15 min, followed by annealing at 100 °C for 10 min. Then, 30 μL of an 8 mg/mL tetraoctylammonium bromide dichloromethane solution is spin-coated onto the perovskite film at 5000 rpm for 30 s and annealed at 100 °C for 5 min. The hole transport material (HTM) solution is prepared, consisting of 0.1 M spiro-MeOTAD, 0.035 M Li-TFSI, and 0.12 M 4-tert-butylpyridine in a 10:1 volume ratio of chlorobenzene (CB)/acetonitrile (ACN). The HTM solution is spin-coated onto the surface at 4000 rpm for 20 s. Finally, a 100 nm thick silver layer is deposited as the metal electrode using vacuum evaporation. This process results in the assembly of FA_0.95_Cs_0.05_PbI_3_ PSCs.

### 3.4. Characterization

The amount of Pb was analyzed using inductively coupled plasma optical emission spectrometry (ICP-OES, ICAP7600, Therom Fisher Scientific, Waltham, MA, USA). A scanning electron microscope (SEM, Phenom Particle X TC, Therom Fisher Scientific, Waltham, MA, USA) was used to observe the surface morphology of the samples and films. X-ray diffraction (XRD, DX-2800 X, Haoyuan, Liaoning, China) was used to analyze the phases of the samples and films. An ultraviolet and visible spectrum (UV-vis, Cary60, Agilent, Palo Alto, CA, USA) was used to study the absorption intensity of the thin films. Dynamic light scattering (DLS, Litesizer 500, Anton Paar, Graz, Austria) was used to analyze the particle size distribution in the solution. Steady-state photoluminescence (PL, DeltFlex, Horiba Scientific, Kyoto, Japan) and time-resolved photoluminescence (TRPL, DeltFlex, Horiba Scientific, Kyoto, Japan) were performed to investigate the carrier dynamics. The J-V characteristics of the PSCs were measured using a Keithley 2401 source meter under an AM1.5G solar simulator (3A Solar Simulator, Enlitech, Shanghai, China).

## 4. Conclusions

The photovoltaic performance of perovskite devices was extensively examined in relation to varying trace amounts of Pb(OH)I. A novel, cost-effective method for synthesizing high-stoichiometry PbI_2_ was developed through the modification of operational steps and the addition of hypo-phosphorous acid, which synergistically inhibited Pb(OH)I formation. The presence of trace Pb(OH)I was found to induce significant alterations in the morphology and crystallinity of the PbI_2_ raw material. These changes subsequently affected the composition of the prenucleation clusters in the precursor solution—resulting in an increased nucleation barrier—hindered the formation of intermediate phases, and promoted the transition to the δ-phase. Films prepared from PbI2 containing Pb(OH)I exhibited reduced grain size and crystallinity as stoichiometry decreased. Pb(OH)I adsorption on the film surface caused structural distortion, leading to δ-phase transition and increased non-radiative recombination. The suppression of Pb(OH)I was observed to improve carrier dynamics and enhance carrier mobility. This improvement was evidenced by the increase in the average carrier lifetime of perovskite films from 127 ns to 229 ns. Photovoltaic devices fabricated using high-stoichiometry PbI_2_ maintained excellent PCE. The highest device efficiency achieved was 22.315%, which is comparable to devices manufactured with commercial high-purity PbI_2_.

## Figures and Tables

**Table 1:** Performance parameters of champion devices in PSCs.

Sample	*Voc* (V)	*Jsc* (mA/cm^2^)	FF (%)	PCE (%)
Commercial PbI_2_	1.03	26.38	82.07	22.295
PbI_1.956_	0.99	26.33	80.84	21.075
PbI_1.932_	0.98	23.95	72.29	16.976

**Table 2:** The stoichiometry of the low-cost PbI_2_ sample and the amount of Pb.

Lead Iodide Sample	Ice Acetic Acid Concentration	Additives	Ratio of I:Pb	Amount of Pb (wt%)
Water-based synthesis method	6.9%	H_3_PO_2_	1.995	46.001

## Data Availability

The data are contained within the article.

## Supplementary Materials

The following supporting information can be downloaded at: https://www.mdpi.com/article/10.3390/molecules29163810/s1, Figure S1: (a) Graph of PbI_1.880_ dissolved in DMF solution; (b) graph of different stoichiometries of PbI_2_ dissolved in DMF solution. From left to right, the stoichiometries (purity) of PbI_2_ are 99.999%, x = 1.995, 1.971, 1.956, 1.942, and 1.932. Figure S2: Schematic diagram of color change in solution during Pb^2+^ titration process. Figure S3: SEM images of PbI1.880. The red circle indicates the needle-shaped crystal structure of Pb(OH)I. Figure S4: (a) Preparation of black-phase perovskite thin films; (b) yellow-phase perovskite thin films undergoing phase transition. Figure S5: XRD-amplified patterns of perovskite thin films prepared from commercial PbI_2_ and different stoichiometric PbI_2_. The peak indicated by the asterisk is the characteristic peak of PbI_2_ impurity. Figure S6: XRD-amplified patterns of perovskite thin films prepared from commercial PbI_2_ and PbI_1.995_. The peak indicated by the asterisk is the characteristic peak of PbI_2_ impurity. Table S1: Pb content of different stoichiometric PbI_2_ measured by ICP-OES.
