# Supplementary material for: The Effect of Component Defects on the Performance of Perovskite Devices and the Low-Cost Preparation of High-Purity PbI2
Source: Molecules. 2024 Aug 11;29(16):3810. doi: 10.3390/molecules29163810 (PMC11357023; doi:10.3390/molecules29163810)
Supplement: Supplementary file 1 [file molecules-29-03810-s001.zip › molecules-3119852-supplementary.pdf]

Supporting Information:

The effect of component defects on the performance of perovskite devices and low cost preparation of high-purity PbI_2

Boyu Dong [1], Yuhan Xie [1] and Yongbing Lou [1,*]

[1] School of Chemistry and Chemical Engineering, Southeast University, Nanjing 211189, China
* Correspondence:lou@seu.edu.cn (Y. L.)

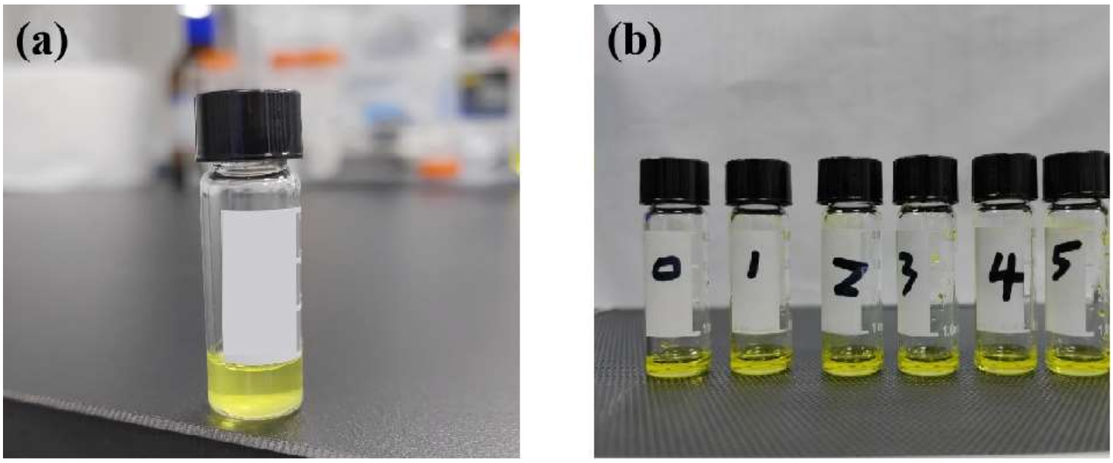

Figure S1. (a) The graph of $PbI_{1.880}$ dissolved in DMF solution; (b) The graph of different stoichiometries of PbI_2 dissolved in DMF solution, from left to right, the stoichiometries (purity) of PbI_2 are 99.999%, x=1.995, 1.971, 1.956, 1.942, and 1.932.

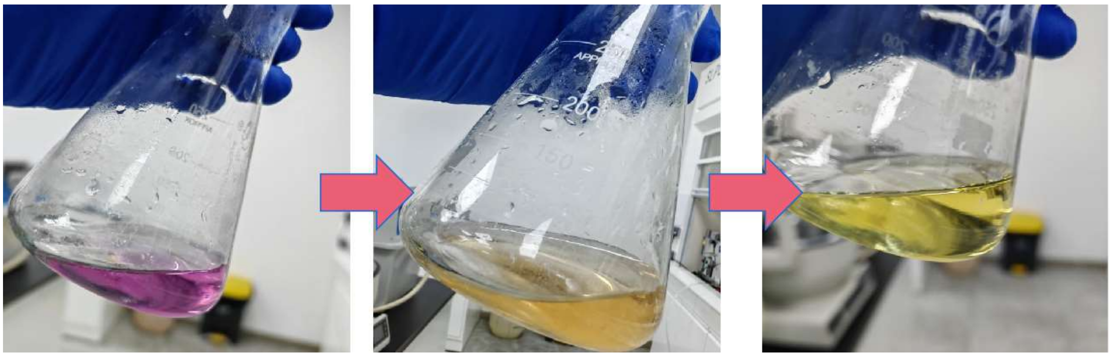

Figure S2. Schematic diagram of color change in solution during Pb^{2+} titration process.

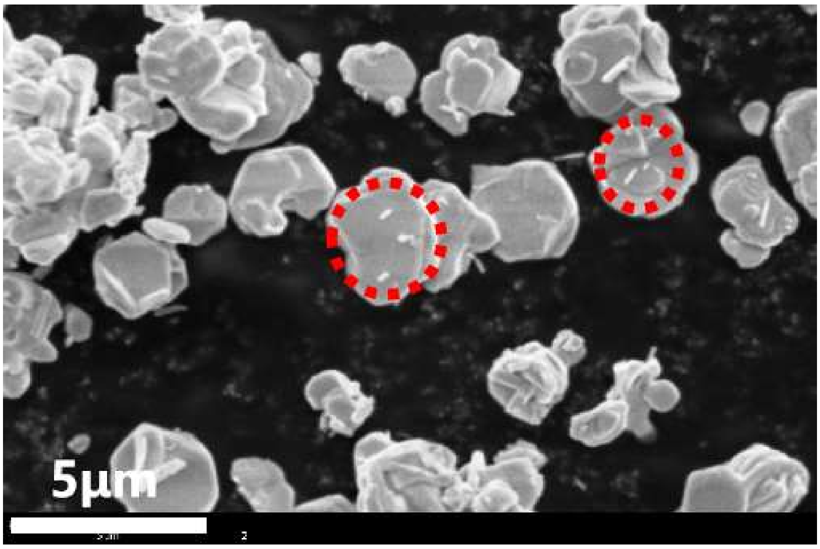

Figure S3. SEM images of $PbI_{1.880}$. The red circle indicates the needle shaped crystal structure Pb(OH)I.

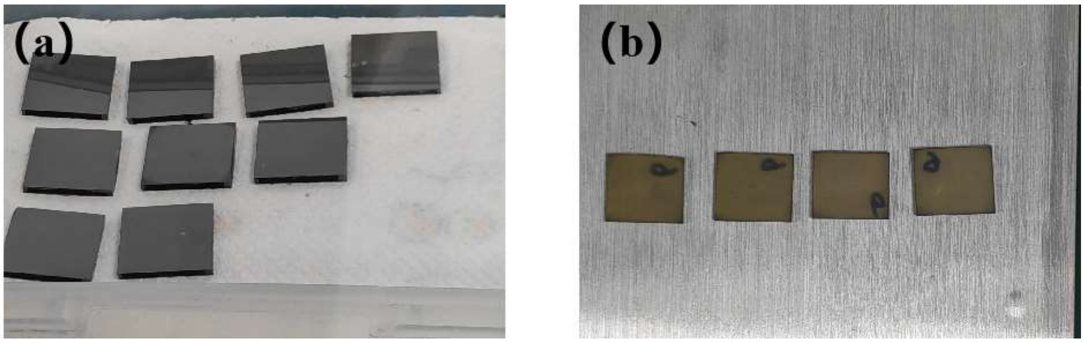

Figure S4. (a) Preparation of black phase perovskite thin films; (b) Yellow phase perovskite thin films undergoing phase transition.

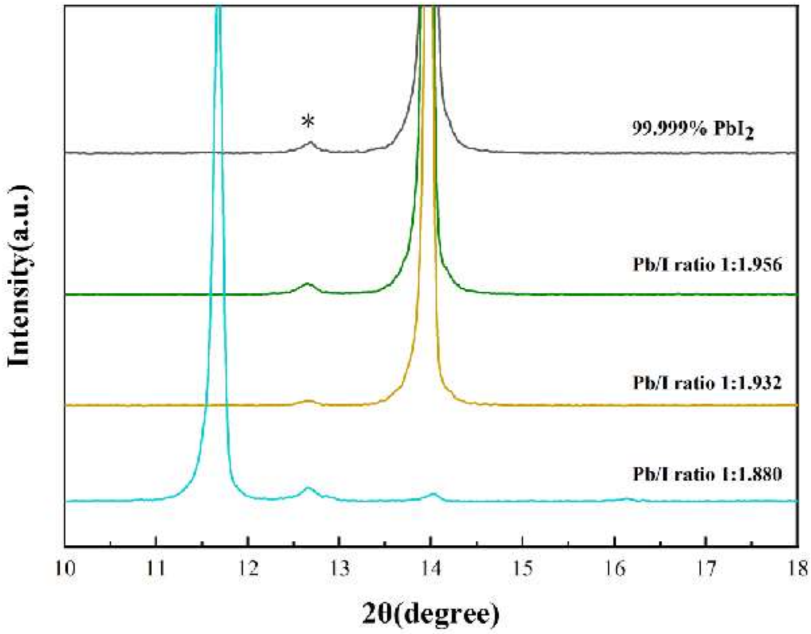

Figure S5. XRD amplified patterns of perovskite thin films prepared from commercial PbI_2 and different stoichiometric PbI_2. The peak indicated by the asterisk is the characteristic peak of PbI_2 impurity.

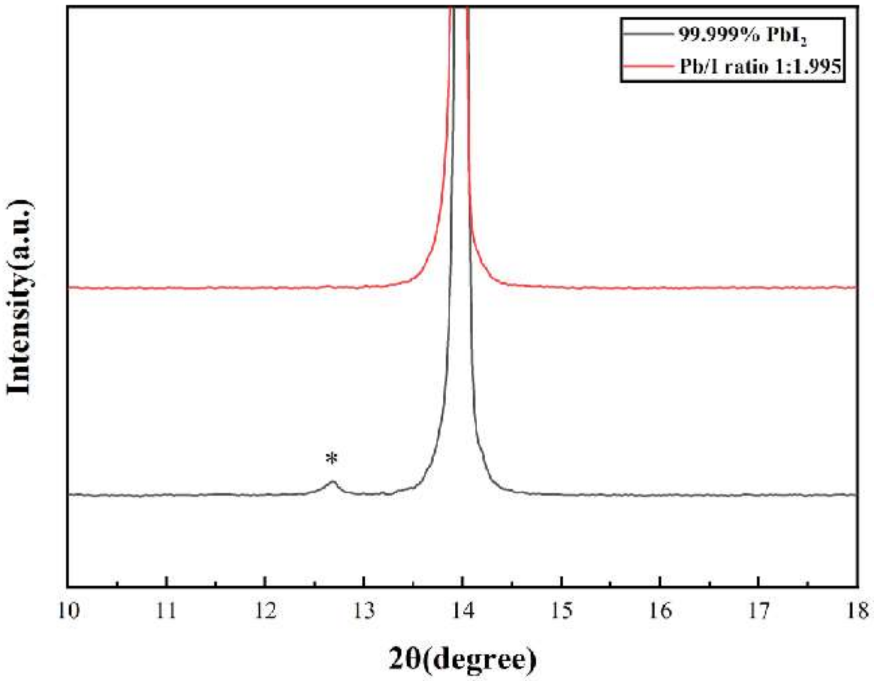

Figure S6. XRD amplified patterns of perovskite thin films prepared from commercial PbI_2 and $PbI_{1.995}$. The peak indicated by the asterisk is the characteristic peak of PbI_2 impurity.

Table S1. Pb content of different stoichiometric PbI_2 measured by ICP-OES.

Lead iodide sample	The ice acetic acid concentration	Amount of Pb(wt%)
$PbI_{1.956}$	5.6%	46.748
$PbI_{1.932}$	2.6%	47.070
$PbI_{1.880}$	1.4%	47.228
